# P03-023 – Autoinflammatory diseases database in Japan

**DOI:** 10.1186/1546-0096-11-S1-A221

**Published:** 2013-11-08

**Authors:** T Kawai, R Nishikomori, M Awaya, K Nakagawa, K Izawa, T Yasumi, O Ohara, T Heike

**Affiliations:** 1 Department of Pediatrics, Kyoto University Hospital, Kyoto, Japan; 2 Research Center for Allergy and Immunology, RIKEN Institute Yokohama Research Promotion Division, Yokohama, Japan

## Introduction

In recent years, responsible genes for autoinflammatory diseases have been increasingly known and clinical phenotype-genotype correlations of these diseases have been explored through international clinical databases such as EUROFEVER project. However, clinical features of genetic disorders could be affected by countries and races of the patients. Actually, patients with Familial Mediterranean fever in Japan show the different distribution of both clinical features and *MEFV* mutations. Up to now, however, no nationwide research of autoinflammatory diseases with standardized contents which can integrate with data of other international studies has been performed in Japan.

## Objectives

We have established autoinflammatory diseases database in Japan. In this research, we designed this database for integration with that of EUROFEVER project in cooperation with Pediatric Rheumatology international trials organization. This integration will enable us to evaluate clinical features and genotypes of autoinflammatory diseases patients in Japan in comparison with the European patients, which will provide further evidences of these diseases, leading to appropriate diagnosis and treatment for the affected patients.

## Methods

Most of diagnoses of the patients with autoinflammatory diseases in Japan have been performed in the central specialized hospitals. In this research, we have collaborated all these hospitals and will collect the patients diagnosed as autoinflammatory diseases through these hospitals. In addition to the diseases devoted to EUROFEVER project, other candidate diseases such as Nakajo-Nishimura like syndromes are included. Inclusion criteria and database contents of the patients are designed by reference to that of EUROFEVER project. Patients’ information will be obtained from collaborated hospitals and the hospitals which actually will treat the patients. Before participation of this study, each collaborated hospital conformed to institutional review board of each hospital and the Declaration of Helsinki.

## Results

Pilot study using paper-based registrations and questionnaires has been closed, and this analysis is ongoing. Now this research is in the transition to web-based system. We hope to show these results in Autoinflammation 2013.

## Conclusion

We have established autoinflammatory diseases database in Japan. This research is ongoing now.

## Disclosure of interest

T. Kawai Grant / Research Support from: the Japanese Ministry of Health, Labor and Welfare, R. Nishikomori Grant / Research Support from: the Japanese Ministry of Health, Labor and Welfare, M. Awaya: None declared, K. Nakagawa Grant / Research Support from: the Japanese Ministry of Health, Labor and Welfare, K. Izawa Grant / Research Support from: the Japanese Ministry of Health, Labor and Welfare, T. Yasumi Grant / Research Support from: the Japanese Ministry of Health, Labor and Welfare, O. Ohara Grant / Research Support from: the Japanese Ministry of Health, Labor and Welfare, T. Heike Grant / Research Support from: the Japanese Ministry of Health, Labor and Welfare

